# Fifth Annual Conference on New and Re-Emerging Infectious Diseases 

**DOI:** 10.3201/eid0809.020279

**Published:** 2002-09

**Authors:** Roberto Docampo

**Affiliations:** ** **University of Illinois at Urbana-Champaign, Urbana, Illinois, USA

## Abstract

The fifth annual Conference on New and Re-Emerging Infectious Diseases was hosted on April 18–19, 2002, by the College of Veterinary Medicine, University of Illinois at Urbana-Champaign (UIUC). The conference featured 8 speakers and 35 poster presentations.

## Recent Infectious Diseases

Beatrice Hahn (University of Alabama, Birmingham, AL) opened the conference with a presentation on the search for the origins of HIV. The evidence indicates that two simian immunodeficiency viruses (SIV), one from chimpanzees (SIVcpz) and the other from sooty mangabeys (SIVsm), crossed the species barrier to humans, generating HIV-1 and HIV-2, respectively. Dr. Hahn stressed the importance of characterizing the prevalence, geographic distribution, and genetic diversity of naturally occurring SIV infections to investigate whether humans continue to be exposed to SIV and if such exposure could lead to additional zoonotic transmissions.

William Hueston (University of Minnesota, St. Paul, MN) gave a personal account of how bovine spongiform encephalopathy (BSE or mad cow disease), appeared in Europe and how chronic wasting disease (CWD,—another transmissible spongiform encephalopathy that affects elk and deer) is spreading across North America. The disease seriously affects the elk industry. CWD causes emaciation and eventually death. The disease has been endemic for decades in elk and wild deer populations in southeastern Wyoming, northeastern Colorado, and a small part of Nebraska. That infections on elk farms could spread the disease to wild populations of elk and deer is of concern and may affect the hunting industry, especially in eastern states, which have large populations of white-tailed deer.

## Bioweapons

Edward Eitzen (U.S. Army Medical Research Institute of Infectious Diseases [USAMRIID] Fort Detrick, MD) recounted the history of state-sponsored biological weapons programs and the emergence of bioterrorism by non-state participants in recent years. The various ways biological agents can be used as weapons and the potential routes of exposure were discussed as prelude to the medical effects of these agents and their effects on the health-care system. Medical countermeasures and other important responses to attacks with biological agents were highlighted, including priorities for the nation to be better prepared. After the anthrax attacks in Florida, New York, New Jersey, and Washington, DC, the threat of biological warfare became much more real; however, these attacks were not the first in the United States. Dr. Eitzen described USAMRID’s role in detecting contaminated material and helping in the clean-up effort in the response to the anthrax attack.

## Cholera and Multidrug-Resistant Tuberculosis 

A filamentous bacteriophage (CTXf) integrated in the *Vibrio cholerae* chromosome encodes the cholera toxin, and Matthew Waldor (New England Medical Center, Boston, MA) described another phage (RS1) that flanks the CTXf prophage in the bacterial chromosome and is important for the CTXf prophage propagation. RS1 relies upon CTXf–encoded proteins for packaging and secretion of its genome; however, RS1 is not simply a parasite, as it can aid the CTXf prophage while exploiting it. The unique RS1-encoded protein RstC is an antirepressor that counteracts the activity of the CTXf repressor, RstR. RstC and RstR appear to form intracellular aggregates that prevent the repressor from binding to its operators. Inactivation of RstR results in increased transcription of CTXf genes and increased transmission of both RS1 and CTXf.

Tuberculosis remains a major global health burden: an estimated one third of the world is infected with *Mycobacterium tuberculosis*. The successful spread of this slow-growing airborne bacteria continues to be a public health challenge. Current problems are further complicated by the rise of multidrug-resistant (MDR) strains, the failure to develop new anti-mycobacterial drugs, and the deadly marriage between HIV and TB. Two genotyping networks have generated data on >60,000 clinical isolates of *M. tuberculosis*, as indicated by Barry N. Kreiswirth (Public Health Research Institute, New York, NY). Unique genetic markers were identified to distinguish various branches of the *M. tuberculosis* genetic lineage that are associated with large MDR outbreaks, such as the drug-resistant W strain that spread through New York State prisons and New York City hospitals. Outbreaks with related clones were also identified in regions of the United States, Russia, South Africa, and in several Asian counties. Preliminary evidence indicates that members of this lineage grow better in macrophage cell lines and are hypervirulent, causing early death in immunocompetent mice.

## Re-Emerging Parasitic Diseases

Through vigorous efforts made in the past two centuries, public health workers have succeeded in developing vaccines, antibiotics, and chemotherapeutics, and as a result most infectious diseases have been brought under control in industrialized countries. However, in developing countries, infectious diseases have been harder to contain, and the increase in migration and movement of populations in the last two decades has made national boundaries disappear as far as the transmission of infection is concerned. Some diseases, such as malaria, have been eradicated from industrialized countries mainly through extensive work on vector control, but their presence in developing countries has increased because of neglect or drug resistance. Donald Goldberg (Washington University, St. Louis, MO) showed how the Malaria Genome Project helped to find novel proteases, several of which appear to function in hemoglobin metabolism. One of these proteases (Histidine Aspartic Protease, HAP) is homologous to three other aspartic proteases involved in hemoglobin metabolism but has a histidine in place of one of the two aspartic acids involved in catalysis. Despite this change, HAP is an active protease with distinct properties, and together with a series of cysteine and metalloproteases and a dipeptidyl peptidase, provides attractive focus for antimalarial drug development.

African trypanosomiasis has reached epidemic proportions in recent years, and its etiologic agents have become noteworthy among molecular biologists for their ability to use genomic rearrangements to change their major surface protein (variant surface protein, VSG). John Donelson (University of Iowa, Iowa City, IA) reviewed antigenic variation in *Trypanosoma brucei* and recent findings on the use of an extranucleolar body containing RNA polymerase I to regulate expression of VSG genes. Donelson provided examples of how the African Trypanosome Genome Project has helped to elucidate the sequence of new expression site-associated genes (ESAGs) and how the RNA interference technique has contributed to identifying essential roles of ESAGs in the *T. brucei* life cycle.

Leishmaniasis has recently emerged as an opportunistic infection after the advent of the AIDS epidemic. Kwang-Poo Chang (Chicago Medical School, Chicago, IL) proposed a hypothetical model to account for nontoxigenic microbial virulence involving two groups of different molecules of parasite origin. One group is responsible for invasion and evasion of mammalian hosts by *Leishmania* to achieve infection but does not directly cause the disease or virulence phenotype. For example, repeated injections of animals with these molecules, such as gp63 and LPG, do not result in any visible disease signs. The other group is formed by immunoreactive epitopes whose interactions with the host immune system result in immunopathology, accounting for the clinical symptoms. The proposed examples include B-cell epitopes specific to *Leishmania* identified by a number of laboratories working with visceral *Leishmania* spp. All have been found in the parasite's cytoplasm often as complex proteins, i.e., ribosomes, nucleosomes (histones), chaperonines, structural proteins (tubulins, kinesin), glycosomes (triose phosphate isomerase). These epitopes are unique to *Leishmania* molecules not shared with those found in autoimmune diseases. One example is the anti-K39 antibodies in kala-azar. K-39 is referred to as 39 aa repetitive peptides found in a kinesin-like gene (5 kb), which is expressed only by the amastigotes of visceral *Leishmania* spp. The anti-K39 antibodies in Indian kala-azar, for example, reach a titer as much as 1:1,000,000. This antibody and other specific anti-*Leishmania* antibodies are nonprotective, as they cannot reach the intracellular antigens within amastigotes inside macrophages but contribute to the hematologic disorders of kala-azar, such as albumin: immunoglobulin G ratio reversal, and hyperplasia of B-cell populations in the lymphoid organs. 

The poster section highlighted recent work on several emerging infectious diseases. Using a cell line in which tubulin was labeled with the fluorescent indicator green fluorescent protein (GFP) and immunofluorescence assay, G.W. Gant Luxton and Kevin Tyler (Northwestern University [NWU], Chicago, IL) demonstrated a rapid association of host tubulins with the plasma membrane at the site of *Trypanosoma cruzi* contact. Juan Leon, from the same laboratory (NWU), showed that captopril, an antifibrotic angiotensin-converting enzyme inhibitor, is effective in ameliorating experimental autoimmune myocarditis and experimental Chagas heart disease. Ileana Cuevas from the Daniel Sanchez Laboratory (University of General San Martin, San Martin, Argentina) reported the presence of a farnesylated protein tyrosine phosphatase in *T. cruzi*. Carlos Lopez-Estraño (NWU) reported experiments using truncated versions of histidine-rich protein II with green fluorescent protein to investigate how they are transported to the malaria-infected erythrocyte cytosol.

*N*-acetylglucosamine-1-phosphate transferase gene is a potential marker for genotyping Old World *Leishmania* isolates, as reported by Kayoko Waki (Chicago Medical School, Chicago, IL). A thioredoxin and a thioredoxin-glutathione reductase are present in *Schistosoma mansoni*, as reported by David Williams (Illinois State University, Normal, IL). Thioredoxin is present in egg-secretory products and is a novel B-cell antigen in schistosome-infected mice. Schistosomes appear to be the first example of an organism with a redox system based exclusively on thioredoxin-glutathione reductase.

Ibulaimu Kakoma's (UIUC, Urbana) and Byeong-Kirl Baek's (Chonbuk, Korea) laboratories reported the use of polymerase chain reaction to verify vertical transmission of *Theileria sergenti* in cows, an important problem for the control of theileriosis. Kakoma and Baek also reported the characterization of the protective response in rats against homologous challenge infections with *Strongyloides venezuelensis*. Finally, Anna M. Schotthoefer (UIUC, Urbana) reported that infection with the larval trematode *Ribeiroia ondatrae* could be responsible for limb malfomations in tadpoles and could explain the observed increase in the frequency of these malformations within natural frog populations. 

In summary, this interdisciplinary conference generated stimulating discussions on various aspects of emerging and re-emerging infectious diseases. Since two SIV viruses were the cause of AIDS in humans, other SIVs can potentially infect humans and cause disease. The spread of chronic wasting disease from elk farms to wild populations of elk and deer in North America is of great concern. Genetic markers have been a great resource to identify MDR strains of *M. tuberculosis*. The interaction of the cholera toxin-encoding phage with an additional phage in the genome of *V. cholerae* has unexpected consequences for their transmission. Non–state-sponsored bioterrorism has changed our appreciation of bioweapons. Parasitic diseases, such as malaria, African trypanosomiasis, and leishmaniasis have re-emerged in recent years and the study of their agents has provided potential targets for their chemotherapy and insights into microbial virulence.

